# Resilience and regeneration for a world in crisis

**DOI:** 10.1007/s13280-025-02287-6

**Published:** 2025-11-25

**Authors:** Joern Fischer, Steffen Farny, Manuel Pacheco-Romero, Carl Folke

**Affiliations:** 1 Social-Ecological Systems Institute, School of Sustainability, Leuphana University of Lueneburg, Luneburg, Germany; 2 Institute of Management & Organization, School of Management & Technology, Leuphana University Luneburg, Luneburg, Germany; 3 Andalusian Centre for Global Change (ENGLOBA), Department of Biology and Geology, University of Almeria, Almeria, Spain; 4 Beijer Institute, Royal Swedish Academy of Sciences, and Stockholm Resilience Centre, Stockholm University, Stockholm, Sweden

**Keywords:** Regenerative design, Regenerative lens, Regenerative sustainability, Seeds of a good Anthropocene, Transformability, Transformation

## Abstract

Both resilience and regeneration are relevant concepts in sustainability science. Resilience thinking has led to improved understanding of cross-scale cycles of growth and renewal, regime shifts, and planetary boundaries. Regeneration highlights the role of positive, place-based and partially self-perpetuating social-ecological dynamics and seeks to foster mutualistic relationships between human and more-than-human entities. This paper lays out similarities, differences and overlaps between work on resilience and regeneration. The concept of regeneration emerged both independently of resilience as well as playing a role within resilience scholarship. We show that the literatures on resilience and regeneration have elaborated complementary ideas and can be combined to derive guidance for improved governance of social-ecological systems. Because of its explicit and proactive future-orientation, the concept of regeneration could help boost nascent efforts to enact biosphere stewardship and develop positive visions for how to re-build a world that is dominated by regenerative rather than degenerative dynamics.

## Introduction

The broad scholarly field of resilience thinking has made central contributions to sustainability science (Folke et al. 2002, 2021; Carpenter et al. 2012). Among the many insights arising from it are the notion that humans and other entities on planet Earth are closely intertwined (Reyers et al. 2018), the concept of regime shifts to describe alternative stable states in social-ecological systems (Scheffer et al. 2001) and, based on that, the delineation of planetary boundaries that define a safe operating space for humanity (Rockström et al. 2009). According to the latter, Holocene-like conditions can only be maintained if the resilience of the Earth system is not fundamentally altered by human activities (Steffen et al. 2018). Resilience thinking thus emphasizes the intertwined fate of humanity and the remainder of the planet. This recognition, in turn, has numerous conceptual and normative implications, leading for example to an increasingly relational understanding of social-ecological complexity (West et al. 2020) and to calls for proactive biosphere stewardship (Chapin et al. 2010; Österblom et al. 2022).

While resilience thinking has been a central tenet of sustainability science for decades, a second body of literature on the concept of ‘regeneration’ has evolved in parallel, but so far, with less influence. Interest in regeneration has multiple origins and dates back several decades (Buckton et al. 2023) but most prominently gained traction via work on regenerative development and design (Reed 2007). Regeneration was framed as an ambition to move beyond doing no harm towards developing synergies between humans and other entities inhabiting Earth, especially in a context of urban design (Mang and Reed 2012, 2020). Work since then has gone as far as to suggest that “regenerative” could be “the new sustainable” (Gibbons 2020) but with a stronger focus on positive outcomes, agency and mutualistic relationships between human and more-than-human entities (Wahl 2016; Buckton et al. 2023). Presently, interest in regeneration appears to be rising in numerous fields, including sociology (Tàbara 2023), economics (Fath et al. 2019) and management (Hahn and Tampe 2021). Just like resilience thinking, the concept of regeneration could yield valuable insights for the stewardship of multi-sectoral social-ecological systems (Buckton et al. 2023; Fischer et al. 2024).

In this paper, we explicitly investigate emerging ideas around the regeneration concept in relation to the well-established field of resilience. Understanding the relationship between these two bodies of work is essential in a context of rising prominence of regeneration. It can help to avoid duplication or re-invention of ideas that are already well-established in resilience thinking, as well as to carve out the added value of the concept of regeneration for sustainability science. Our perspective article should be seen as an entry point to conversations about the relationship between resilience and regeneration. We acknowledge that our positionality has inevitably shaped our understanding and the arguments we present (Yip 2024). Most notably, we have historically been more deeply involved in the resilience discourse than in the regeneration discourse; we are most strongly versed in social-ecological literature; and our insights largely stem from English-language scientific literature. All of these points inevitably limit our ability to fully capture the diversity of meanings embodied by regeneration.

We first sketch out the origins and development of both resilience and regeneration. We then investigate how established concepts in the body of work on resilience relate to evolving ideas in the growing body of literature on regeneration. Based on this understanding, we provide an outlook on how regeneration could shape sustainability science and practice in the future—by helping to enact biosphere stewardship, as well as by informing future-oriented responses to social-ecological perturbations that humanity will undoubtedly face within the coming decades.

## Origins and development of resilience and regeneration

**Fig. 1:**
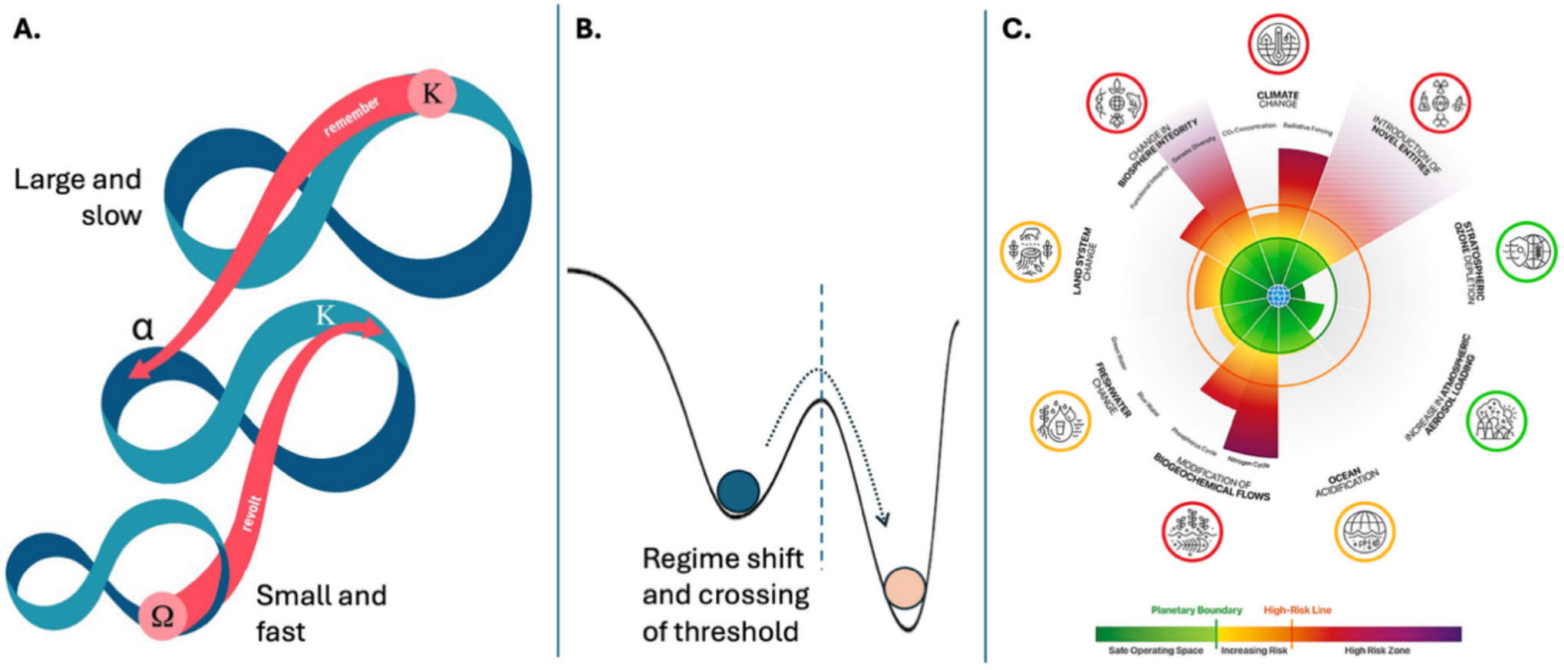
Illustrative examples of three key ideas from resilience thinking. **A** The Panarchy model recognizes that interlinked systems at multiple spatiotemporal scales undergo cycles of collapse and renewal (figure adapted from Biggs et al. 2021). **B** The ball-in-cup metaphor is a widely used way to visualize the concepts of alternative stable states, thresholds, and regime shifts (original drawing). **C** Planetary boundaries refer to thresholds for different drivers of change in the Earth System (figure adapted from Planetary Boundaries Science (PBScience) 2025)

Resilience has its roots in the Latin word ‘resilire’, which broadly means to bounce back. Beyond specific definitions of resilience—which differ between engineering and systems ecology, for example (Holling 1996)—within a sustainability context, the most prominent contemporary use of resilience is about capacities to live and thrive with change in a context of social-ecological complexity. This school of thought emerged from observations that natural ecosystems are characterized by cycles of stability and perturbation that span multiple spatial and temporal scales (Holling 1973, 1992, 2001). The classic depiction of this behaviour is via the panarchy model; that is, nested cycles of system change that each involve phases of growth, conservation, release and reorganization (Holling 2001) (Fig. 1a). The ability of a system to continue functioning in the long term—its resilience—arises from the interplay of exogenous drivers and disturbances, as well as endogenous conditions of the system (Folke et al. 2004). One phenomenon that attracted particular attention was that different types of ecosystems could quickly and unexpectedly ‘flip’ from one apparently stable state to another (often less desirable) state. Such regime shifts occur when thresholds in critical drivers are crossed and when the endogenous conditions within a system have undermined its resilience (Folke et al. 2004). Regime shifts have been observed in diverse types of ecosystems, including coral reefs, savannahs, and freshwater lakes (Scheffer et al. 2001) (Fig. 1b).

By the 2000s, resilience was considered not only for ecosystems but also for complex social-ecological systems (Berkes et al. 2003; Folke 2006)—that is, for situations where human and more-than-human entities interact in complex ways and together shape the overall functioning of the system. “Resilience thinking” was coined as an overarching perspective during this time to describe a way of approaching social-ecological systems that acknowledges complexity, including social-ecological relationships and feedbacks, resilience, alternative stable states, as well as the potentials for adaptation and transformation (Walker and Salt 2006). The critical role of governance for shaping social-ecological outcomes was also increasingly recognized during this time (Folke et al. 2005; Lebel et al. 2006; Anderies et al. 2006); most famously by connecting with Elinor Ostrom’s framework (Ostrom 1990) on governing common pool resource systems (Berkes and Folke 1998; Ostrom 2009). Based on that work, polycentric governance systems—where decisions are made by not just one central actor, but rather by multiple, partly interconnected actors at different levels of a system—are now widely believed to facilitate resilience.

An additional critical contribution of resilience thinking is that it conceptually underpins the delineation of planetary boundaries (Rockström et al. 2009) (Fig. 1c)—the basic idea being that regime shifts could challenge the safe operating space for humanity on Earth if thresholds in various drivers of change are transgressed (e.g. land use change, climate change and others) (Rockström et al. 2009; Steffen et al. 2015; Richardson et al. 2023; Planetary Boundaries Science (PBScience) 2025). Not least through the planetary boundaries framework, resilience has now become a mainstream concept in sustainability science.

Modern work framed around resilience typically emphasizes the importance of active biosphere stewardship (Steffen et al. 2011; Österblom et al. 2022; Chapin et al. 2022), and often emphasizes the need for transformative social-ecological change (Folke et al. 2021). Beyond its roots in ecology, resilience has also become a central concept in multiple other disciplines, including sociology (Aldrich and Meyer 2015; Carmen et al. 2022), psychology (Luthar and Cicchetti 2000; Fletcher and Sarkar 2013), management and economics (Barthel and Isendahl 2013; Linnenluecke 2017), as well as speaking directly to policy development (Brown 2016; Rockström et al. 2023). Here, resilience has been variously considered as a trait, a capacity, or a set of practices (and processes) of the human component of systems, from individuals to organizations or communities (see Folke 2016 for an extensive review). Slowly a shift is emerging in these fields, too, to acknowledge that social dynamics are embedded within and reciprocally related with ecological dynamics as part of the biosphere.

Regeneration also has Latin roots, namely in the verb ‘regenerare’, which broadly means to create again. The most prominent regeneration discourse in a sustainability context originated within a context of urban development and design, where it sought to go beyond less ambitious framings of ‘sustainability’ which implied that doing less harm, or compensating it where possible, were acceptable development goals (Reed 2007). Instead of maintaining a system (‘sustaining’ it), regenerative design sought to foster positive relationships between people, the built environment, and the natural environment, thereby promoting the thriving of both people and nature (Mang and Reed 2020). Like resilience, the term regeneration has been used in many other fields, spanning a substantial range of interpretations from ecological design to a strongly procedural focus on bottom-up engagement of diverse actors (Robinson and Cole 2015). Regeneration and broadly synonymous terms are now found in literature on agriculture (Schulte et al. 2022; Chaplin-Kramer et al. 2023), management (Hahn and Tampe 2021; Konietzko et al. 2023), sociology (Tàbara 2023), and education (Caniglia et al. 2019); and in some cases (e.g. regenerative agriculture) the histories of specific discourses date back to times well before Reed’s (2007) seminal work on regenerative design (an in-depth review of the semantic origins of various terms is beyond the scope of this paper; see Buckton et al. 2023 for a review that provides additional details on the origins and use of regeneration in multiple fields). Noting growing interest in regeneration across multiple fields, Buckton et al. (2023) and Fischer et al. (2024) recently offered cross-disciplinary syntheses of existing work on regeneration. Accordingly, and notwithstanding the diversity of nuances within literature on regeneration (Robinson and Cole 2015), central tenets of a focus on regeneration can be defined as follows.

**Fig. 2:**
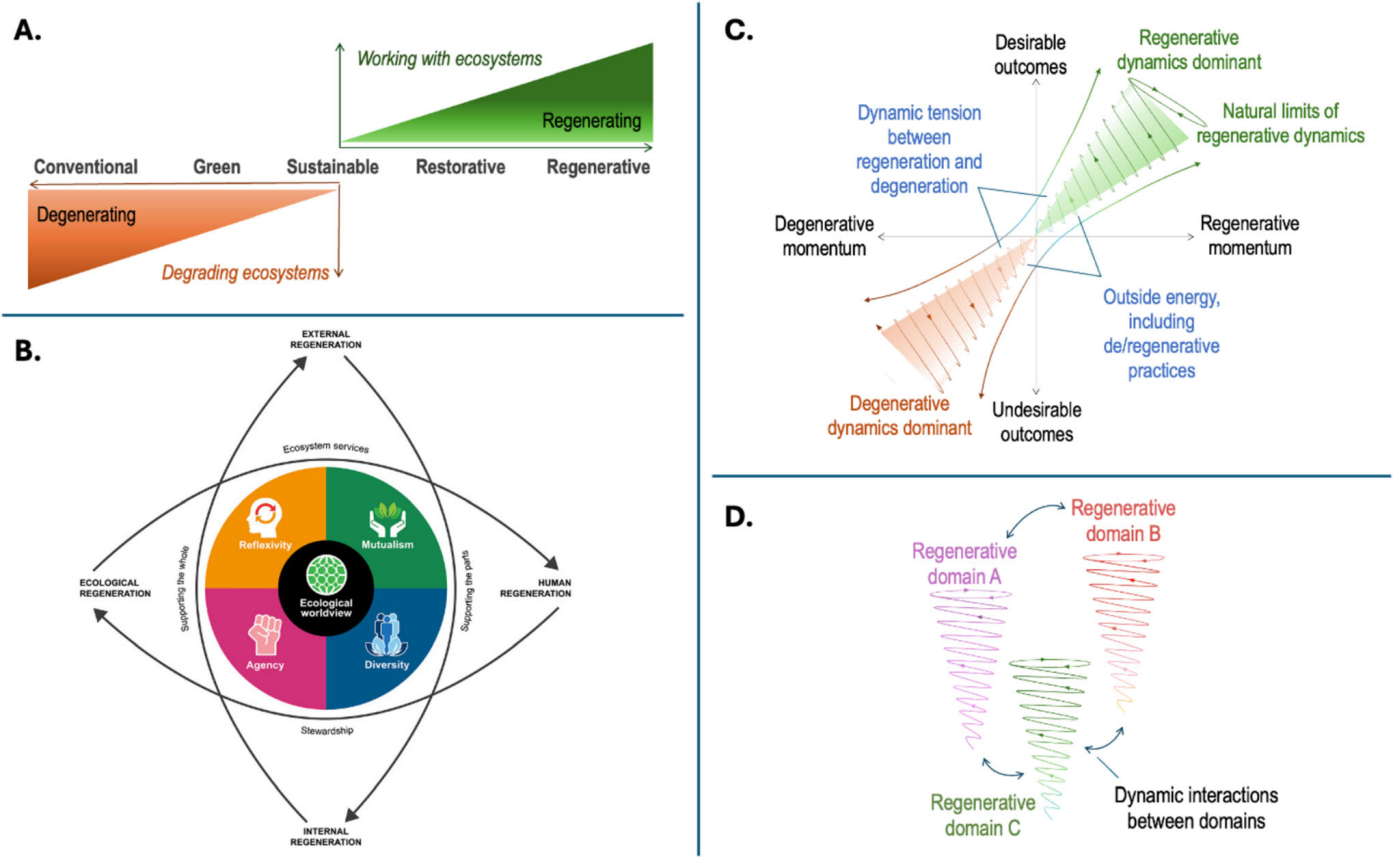
Illustrative examples of four key ideas from regenerative systems thinking. **A** Regeneration seeks to go beyond doing no harm (based on Reed 2007). **B** The ‘regenerative lens’ emphasizes central building blocks of regenerative systems thinking, including agency and mutualistic interactions between humans and other species (see Buckton et al. 2023 for details). **C** Regenerative dynamics can be conceptualized as ‘upward spirals’ of change that are shaped by a system’s regenerative momentum and the energy input into the system (see Fischer et al. 2024 for details). **D** Different regenerative domains can positively influence one another (Fischer et al. 2024)

First, most scholarship on regeneration is explicitly place-based (Camrass 2023) and seeks to promote place-based net improvements in the world (Fig. 2a). The focus thus is on reversing degeneration (instead of only minimizing, mitigating, or compensating it), with an emphasis on the need for and power of human agency to actually bring about such positive changes in system dynamics (Reed 2007; Buckton et al. 2023).

Second, regeneration targets social-ecological wholes, seeking to generate anew their inherent capacity for vitality, viability and social-ecological co-evolution, instead of depleting underlying life support systems and resources (Mang and Reed 2020; Camrass 2023). As such, a regenerative worldview explicitly emphasizes mutualistic interactions between human and more-than-human entities (Buckton et al. 2023) that facilitate the thriving or health of an overall social-ecological system (Fig. 2b).

Third, conceptualizations of regeneration have become increasingly comprehensive—explicitly defining, among others, the processes and dynamics operating within a system, existing degenerative or regenerative momentum within the system, and practices that contribute to either degenerative or regenerative momentum (Fig. 2c, Fischer et al. 2024). Degenerative or regenerative momentum refers to the trajectories of systems tending to be ‘locked in’ on certain pathways, which only change with substantial variation of energy input into the system.

Fourth, regeneration explicitly promises to be an integrative lens to connect multiple domains and scales (Buckton et al. 2023; Fischer et al. 2024). Dynamics in multiple connected sub-systems may interact in positive or negative ways—such that degenerative cascades (leading to a polycrisis (Søgaard Jørgensen et al. 2024)) are, in principle, equally plausible to regenerative cascades resulting from reinforcing dynamics across multiple domains or scales (Fig. 2d).

## Overlaps and differences between resilience and regeneration

Resilience and regeneration are complementary meta-concepts. The rapidly growing bodies of work on these two meta-concepts show certain overlap in scope and key ideas. In the following paragraphs, we highlight conceptual similarities between resilience and regeneration. We then explain how, despite these similarities, the focus of these two bodies of work is subtly different, such that the rise of regeneration is complementary and provides additional nuance to existing insights stemming from resilience thinking (Table 1). In describing the conceptual relationship between resilience and regeneration, we explore how key ideas within literature on regeneration map onto concepts generated under the umbrella of resilience thinking. We step through these links from the perspective of regeneration because (i) the number of formalized ideas associated with ‘regeneration’ is smaller than the number of formalized ideas associated with ‘resilience’; and (ii) although resilience science is continuously developing, it is an established and well understood field, whereas regeneration is still developing.

**Table 1:** Simplified summary of key characteristics of scholarship on resilience and regeneration

	Resilience	Regeneration
Disciplinary use	Multiple decades of use in diverse fields, including physics, engineering, ecology and various social sciences; the most widely known discourse in a sustainability context can be traced back to work on ecosystems (Holling 1973)	Multiple decades of use in diverse fields, including urban design, agriculture, development, ecology and business; the most widely known discourse in a sustainability context is grounded in urban design and development (Reed 2007)
Spatial scales	Nested spatial scales, from local to global; acknowledging complex links across multiple scales	Often primarily local and place-based but cognisant of cross-scalar momentum and dynamics
Temporal scales	Multiple temporal scales of interest; the present being seen in the context of the past and with a view to the future	Inherently futures-oriented
Actors and agency	Diverse actors who can be (self-)governed to reach good or bad outcomes	Local actors and their immediate agency to achieve desirable, ongoing improvements are foregrounded (i.e. regenerative practices)
Prominent themes captured (non-exclusive)	• Maintain functioning despite perturbations or shocks • Systems thinking • Cycles of collapse and renewal across multiple scales (panarchy) • Thresholds and alternative stable states • Planetary boundaries • Polycentric governance	• Net-positive contributions • Holism • Interlinked, partly self-perpetuating ‘upward spirals’ of change • Mutualistic human-nature relations • Co-evolution of humans and more than human nature • Social-ecological health, care and well-being (inner and outer transformations)

*Regenerative dynamics.* Regenerative dynamics occur when a system undergoes repeated events of renewal (Fischer et al. 2024). For example, a forest exhibits regenerative dynamics after a wildfire when it regrows once again into a forest (Gunderson and Holling 2002). Similarly, a social-ecological system exhibiting cross-domain regenerative dynamics may be one in which economic prosperity goes hand in hand with thriving ecosystems and human well-being. In resilience thinking, regenerative dynamics dominate the growth phase within the adaptive cycle (Holling 2001), with regeneration being a combination of the memory as well as novelty of the system (Gunderson and Holling 2002).

*Degenerative dynamics*. Degenerative dynamics are the opposite of regenerative dynamics. They are spirals of deterioration and are, in essence, what is causing the increasingly severe transgression of planetary boundaries (Rockström et al. 2009; Steffen et al. 2015; Richardson et al. 2023; Planetary Boundaries Science (PBScience) 2025). Cross-domain interactions in degenerative dynamics, in turn, result in a polycrisis, where different crises spreading through multiple social and ecological domains feed one another in synergistic ways (Søgaard Jørgensen et al. 2024). For example, global climate change exacerbates biodiversity loss, and this can further exacerbate climate change. Ultimately, interconnected degenerative dynamics can lead to regime shifts and social-ecological traps from where a system cannot easily escape (Lade et al. 2017; Søgaard Jørgensen et al. 2024). Ongoing degenerative dynamics could even lead to cascades of undesirable regime shifts, as has been suggested, for example, in the context of climate change dynamics (Steffen et al. 2018). Degenerative dynamics can be very resilient, in the sense of difficult to break. Hence, resilience may be both useful or harmful. Breaking out of undesired resilience requires agency and action that reduce the resilience of a malfunctioning degenerative system, and tip it, or transform the system into a new development path or trajectory (Chapin et al. 2010; Westley et al. 2011).

*Regenerative momentum*. Provided sufficient initial energy input into a given system, regenerative dynamics can eventually become partly self-perpetuating (noting that the laws of thermodynamics mean that some energy input is inevitably required to maintain regeneration). Regenerative momentum thus means that the initial effort needed to break degenerative dynamics is higher than the effort needed to maintain regenerative dynamics—once such positive dynamics have gained momentum, they can self-sustain (Fischer et al. 2024). The idea of shifting from degenerative dynamics to regenerative dynamics is captured, for example, by the ball-in-cup metaphor—a threshold needs to be crossed for a regime shift to take place, from a system that is dominated by degenerative dynamics to one that is dominated by regenerative dynamics. Once the system is in a domain of regenerative dynamics, these dynamics can build resilience and stabilize the system within this new (more desirable) state (Herrfahrdt-Pähle et al. 2020). Finally, regenerative momentum is also central to what makes the notion of ‘seeds of a good Anthropocene’ enticing (Bennett et al. 2016)—systems with regenerative momentum have the seeds to engender hope and inspiration for the possibility to create systems that can self-perpetuate positive dynamics (Fischer et al. 2024).

*Degenerative momentum*. Degenerative momentum is the opposite of regenerative momentum. It describes a negative path dependency that undermines the resilience of a system, potentially causing one or multiple subsequent regime shifts into undesired (and yet, resilient) stability domains. Examples of rising degenerative momentum include the deployment of technological ‘solutions’ at the expense of the natural environment that only coerce resilience and erode adaptive capacity (such as inappropriate investments in irrigation infrastructure or the short-sighted management of production forests Holling and Meffe 1996; Anderies et al. 2006; Felton et al. 2024). Once regime shifts have taken place from a desired to an undesired stability domain, degenerative momentum can be very high, and the system can end up in a lock-in situation or social-ecological trap (Allison and Hobbs 2004; Carpenter and Brock 2008).

*Regenerative practices.* Although some systems regenerate simply through natural energy input (e.g. sunlight and rain), others need targeted action to kick-start or maintain regenerative dynamics (Fischer et al. 2024). For example, in regenerative agriculture, farmers actively apply certain practices to support production through increasing soil fertility and biodiversity (Schulte et al. 2022). The notion of regenerative practices is closely related to the idea of purposive actions that foster resilience and adaptive capacity, which is captured, for example, by the term of ‘biosphere stewardship’ in resilience thinking (Folke et al. 2021; Österblom et al. 2022).

*Limits to regeneration*. In principle, systems will continue to (co-)evolve, and regeneration thus can play an important role in perpetuity. However, when a particular type of system has reached a highly complex state (e.g. that of an old-growth forest), it may not be able to further ‘improve’ its state despite ongoing regeneration within it, and instead is being maintained at an attainable ‘maximum’ outcome condition (Fischer et al. 2024). This natural limit to growth is recognized in the adaptive cycle as the conservation phase (Holling 2001). In this phase the system tends to become more and more interconnected, and brittle when subject to shocks and disturbances.

*Agency.* Agency is a central idea to regeneration—humans are seen to have the capacity to engender change in systems so that they can become regenerative (Buckton et al. 2023). In resilience thinking, agency is captured implicitly through a series of important concepts. Adaptive capacity describes the ability of actors within a system to adjust their behaviour in order to respond to external stressors (Allison and Hobbs 2004); transformability describes the capacity of a system to be fundamentally re-shaped by the actors within it (Walker et al. 2004); and biosphere stewardship implies proactive (regenerative) behaviour by diverse actors and humanity as a whole (Folke et al. 2021; Österblom et al. 2022). In addition, polycentric governance implies the simultaneous presence of top-down forces or structures and bottom-up agency. The role of actors and agency—including local actors and their connection to specific places—thus has a long tradition in resilience scholarship (Folke et al. 2005b; Brown and Westaway 2011).

*Mutualistic interactions*. Finally, literature on regeneration tends to emphasize the need for mutualistic interactions between human and more-than-human entities within the system (Buckton et al. 2023). This focus is broadly consistent with the fundamental understanding that underpins the notion of a social-ecological system: humans use and care for their environment, which in turn generates vital benefits for humans (Fischer et al. 2015). However, the intertwined nature of human–environment interactions that is meant to be depicted by a social-ecological system is sometimes criticized for implicitly representing dualistic human-nature thinking (Reyers and Bennett 2025). The explicit recognition of mutualistic interactions in literature on regeneration means that regeneration goes beyond traditional work on resilience in this realm, by embracing relational, non-dualistic thinking, which has in fact also been advocated by some resilience scholars in the recent past (West et al. 2020; Reyers et al. 2022). A look beyond the superficial connotation of certain terms thus suggests that there may be little difference in human-nature conceptualisations between the two bodies of literature—both recognize that human and more-than-human entities are closely intertwined.

The preceding discussion shows that resilience and regeneration are interlinked in numerous ways. This overlap should not be surprising because—especially within a social-ecological context—both concepts are rooted in systems thinking. Still, resilience and regeneration are subtly different with respect to which aspects of complex system dynamics they emphasize. Resilience is a system property that is desired primarily for the presence. However, the broader school of ‘resilience thinking’ has always emphasized that dynamic change—including cycles of collapse and renewal—is a necessary precondition for the continued existence of any higher-level system. It would therefore be false to claim that resilience is static or focuses only (or even primarily) on the present (Anderies and Folke 2024). Thus, it might be most accurate to see resilience as speaking strongly to the present, but being informed by the past, and with a view to the future. Indeed, recent work has suggested that resilience can help not only to bounce back, but also to ‘bounce forward’ following major shocks such as the COVID-19 pandemic (Hynes et al. 2020).

Regeneration, in contrast, makes the possibility for positive, ongoing renewal explicit and emphasizes the future potential of systems. The focus is less on averting the collapse of a much-cherished system, but instead on creating something new that is characterized by healthy dynamics from the outset. Through its focus on continuous re-creation, regeneration is a strongly futures-oriented concept by nature. This, in combination with its emphasis on mutualistic human-nature relationships and agency, makes regeneration a valuable complement to resilience. In this sense, regeneration would represent a next practical step on the ground of the transformability pathway of resilience thinking, a way to help revitalize and better prepare a social-ecological system when facing plausible shocks and towards sustainable futures (Folke et al. 2021; Farny and Dentoni 2025). The heuristic value of regeneration may be especially important in an era where many people experience fear because of rising uncertainty (UNDP (United Nations Development Programme) 2022), and where increasingly interconnected perturbations challenge humanity to (re-)build new and more self-sustaining systems at both small and large scales.

## Outlook: Carrying resilience and regeneration into the future

**Fig. 3:**
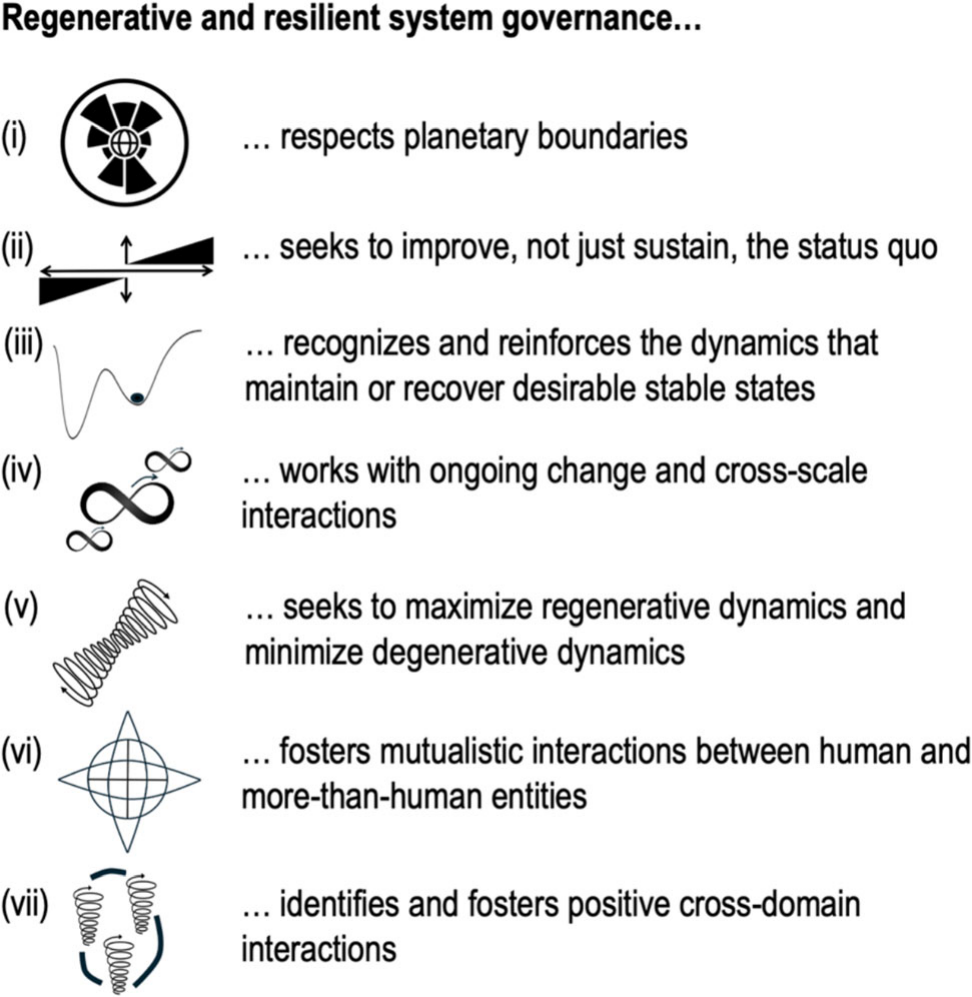
Seven key principles for the regenerative governance of social-ecological systems. The seven principles depicted here draw on the seven central insights of resilience and regenerative systems literature as summarized in Fig. 1 for resilience (i, iii, iv) and Fig. 2 for regeneration (ii, v, vi, vii). While each item is derived from either body of literature on resilience or regeneration, the list of items offered here is fully complementary. See main text for details and references and Table 1 for some of the most pertinent similarities and differences between the two bodies of literature

The key insights from scholarship on resilience and regeneration—as summarized in Figs. 1 and 2, respectively—are complementary. Together, they provide a set of seven simple principles that could be hopeful starting points for proactively governing our world towards a better future (Fig. 3).

Resilience has been one of the most successful boundary objects in sustainability science to date. Although different scholars have held slightly different views of what exactly resilience is (Nimmo et al. 2015), the essence of what is captured by the term ‘resilience’ has appealed to countless scientists and practitioners (Strunz 2012), and arguably, has shaped humanity’s science and action for the better. Resilience thus has become a cornerstone concept for those seeking to understand and improve social-ecological systems. Despite the initial excitement about resilience being some decades in the past, the core insights of resilience thinking will remain deeply relevant for many years to come.

In comparison to resilience, regeneration is the new kid on the block within the mainstream of sustainability science, and it remains to be seen what it can and cannot do. Here, we offer two thoughts on why regeneration could be an especially valuable addition in efforts towards redirecting social-ecological systems towards sustainability.

First, a focus on regeneration can further boost nascent efforts to enact biosphere stewardship, building on the conceptual groundwork of resilience thinking. From resilience thinking, we know, in principle, how to keep systems from flipping into undesirable states or shift from undesirable to more desirable states (Herrfahrdt-Pähle et al. 2020); we know how to enhance the adaptive and transformative capacity of systems; and tangible “seeds of a good Anthropocene” are being documented and analysed by scholars working on resilience (Bennett et al. 2016). The concept of regeneration can help us now to move forward more boldly in the direction of social-ecological transformations towards biosphere stewardship. Conceptually, regeneration can help through its explicit focus on human agency and mutualistic social-ecological relationships, as well as through the idea that instating positive dynamics in one domain can help to foster positive dynamics in one or more other domains (Fischer et al. 2024). This realization suggests that large-scale, multi-sector systemic improvements are, at least in principle, just as possible as large-scale multi-sector crises. ‘Poly-opportunities’ thus become a conceptual possibility. The emphasis within the regeneration discourse on human agency suggests that it is up to us, as humanity, to learn more about such poly-opportunities and make them central policy goals. How can progressive goals in sectors such as health, environment, economy, education, and agriculture be linked, such that they help to boost one another rather than cause trade-offs? Thinking in regenerative terms, such synergies likely exist but have not been systematically identified to date (Fischer et al. 2024). Signs of such change are happening, like the new measure ‘Gross Ecosystem Product’ applied in China (Zheng et al. 2023) or the recently suggested ‘Nature Relationship Index’ addressing how well people and nature are thriving together (Ellis et al. 2025).

Second, it appears inevitable that humanity will witness increasingly severe perturbations in all kinds of social-ecological systems, at all kinds of scales, over the coming years and decades. Using terminology from resilience thinking, this means that release, reorganization and renewal will be inevitable companions in the years to come; less empowering language has warned of various types of ‘collapse’ (Ehrlich and Ehrlich 2013). With such changes becoming increasingly likely in the future, the time is ripe to actively plan for how to revitalize systems after such changes and how to redirect them towards a better future. It is in this context that the regenerative social-ecological systems approach becomes promising and relevant as a hope-inspiring way forward and as a way for concrete action. Thanks to resilience thinking, we know a lot about what kinds of properties social-ecological systems need to have to endure difficult times; if we add to these properties the mindset of mutualistic relationships between human and more-than-human entities and active attempts to foster positive dynamics both within and across major social-ecological domains, we may be at the beginning of a new vision for how to collectively rebuild our world—starting now.
